# Exploring the adequacy of smoking cessation support for pregnant and postpartum women

**DOI:** 10.1186/1471-2458-13-472

**Published:** 2013-05-14

**Authors:** Tracey Borland, Alexey Babayan, Saeeda Irfan, Robert Schwartz

**Affiliations:** 1 Ontario Tobacco Research Unit, Dalla Lana School of Public Health, University of Toronto, 155 College St, Toronto M5T 3M7, Canada; 2 Centre for Addiction and Mental Health, 33 Russell Street, Toronto M5S 2S1, Canada

## Abstract

**Background:**

Smoking in pregnancy exemplifies the relationship between tobacco use and health inequalities. While difficulty reaching and engaging this population in cessation support is often highlighted in the literature, there is limited research that explores the factors that shape the provision and use of support by this subpopulation. Using Ontario, Canada, as a case study, this study examines how the use of cessation support by women is encouraged or discouraged by cessation policy, programming and practice; how geographical and sociocultural factors influence provision and uptake of support; and how barriers and challenges can be addressed through a comprehensive approach.

**Methods:**

Semi-structured, in-depth interviews with key informants (31) and pregnant or postpartum women (29) were conducted to examine the cessation needs of this subpopulation, barriers to the provision and uptake of cessation support and directions for policy, service provision and programming.

**Results:**

Key barriers included: the absence of a provincial cessation strategy and funding, capacity and engagement/accessibility issues. Geographical features presented additional challenges to provision/uptake, as did the absence of resources tailored to Aboriginal women and adolescents. Key informants recommended a comprehensive cessation strategy to facilitate coordination of cessation resources provincially and locally and elucidated the need for capacity building within tobacco control and within reproductive, child and maternal health. Participants also highlighted the need to further develop tobacco control policies and target the social determinants of health through poverty reduction, housing and education support. The provision of incentives, transportation, childcare and meals/snacks; adoption of woman-centred, harm-reduction and stigma reduction approaches; and promotion of programs through a variety of local venues were recommended by participants to address engagement and accessibility issues.

**Conclusions:**

The current cessation system in Ontario is not equipped to adequately reduce smoking among pregnant and postpartum women. A comprehensive, multi-sector strategy designed to provide tailored and sustainable support through different system entry points is needed. A cultural shift in practice is also necessary to eliminate mixed messaging, strengthen practice and encourage open channels of communication about smoking between women and their providers. The study highlights the need to address smoking among women in a more holistic manner and for capacity building strategies that focus on strengthening providers’ competency and confidence in practice. Future research should explore: capacity building strategies, especially among rural and remote communities; the smoking and cessation experiences of different subpopulations of pregnant and postpartum women; the effectiveness of tailored strategies; and interventions that address smoking among partners and other family members.

## Background

While smoking rates have declined over the past decade, socioeconomic inequalities in smoking persist in high income countries [1-4]. An important public health issue that exemplifies the relationship between tobacco use and health inequalities is smoking in pregnancy. Smoking in pregnancy and the postpartum period is a complex issue that is shaped by a variety of socioeconomic, sociocultural and psychosocial factors. Specific determinants include: low education, low income and low occupational status, young age, Aboriginal status, lone parenthood, smoking in social networks, and mental health, addiction and violence issues [5-9]. Further, the association between disadvantaged circumstances during the fetal, infant and childhood stages, and behaviours such as smoking and/or the emergence of chronic diseases in adulthood is well documented [10-13]. For example, low birth weight and preterm birth are associated with prenatal smoking and are powerful predictors of development and health and disease across the lifecourse [11]. Relapse rates are generally high among this subpopulation [14].

In 1992, the Ontario Tobacco Strategy aimed to eliminate smoking among pregnant women by the year 2000 [15]. However, approximately 1/10 pregnant women in Ontario are current smokers [16]. These rates vary greatly between Ontario’s seven public health regions (34% in the Northwest region to 5.8% in Central region), and have remained relatively unchanged since 2004. Further, current smoking rates among women under the age of 20 is striking; over 40% of pregnant adolescents in five of Ontario’s seven public health regions smoke during pregnancy [17].

Difficulty reaching and engaging this population is frequently highlighted in the literature and might consequently impede the development of tailored programs and services [18,19]. A comprehensive approach, which involves policy, program and practice measures, is recommended to promote the uptake of cessation support among the general population [20]. There is, however, a paucity of literature on how these measures might shape the use and provision of cessation support for pregnant and postpartum women. Existing research has focused primarily on health care provider engagement (mainly physicians, nurses and midwives) in cessation for pregnant women. A recent comprehensive review revealed that less than 50% of providers practiced all components of the 5As with their pregnant patients [21]. Similarly, a qualitative systematic review found limited monitoring, referral and follow-up of women’s smoking status [22]. The authors identified the following barriers and challenges to service provision: low confidence in practice, fear of compromising the patient-provider relationship and the perception that cessation interventions are ineffective for pregnant women. Additional aspects of service delivery that impeded the provision of services included lack of knowledge, discrepancies in provider practice, a judgmental manner of communication, and lack of time, training and organizational cessation protocols. Further, there is a paucity of research that examines how contextual factors, such as geographical and sociocultural issues, influence the provision and use of support [21]. This study addresses gaps in understanding challenges in the provision and uptake of services.

Using Ontario, Canada, as a case study, this study explores how Ontario cessation policy, programming and practice encourage or discourage the provision and uptake of support by women. It further explores geographical and socio-cultural factors related to cessation and how barriers and challenges to provision and access to services can be addressed through a more comprehensive approach. While the provision of cessation programs and services for priority populations is mandated by the Ontario Public Health Standards^a^, there is an apparent lack of cessation support for pregnant and postpartum women across Ontario’s 36 Public Health Units [23]. While this paper focuses upon improving Ontario’s cessation system to better meets the needs of pregnant and postpartum women who smoke, a forthcoming paper explores socio-ecological barriers, as illuminated by women in this study.

## Methods

### Design, approach and ethical procedures

Semi-structured, in-depth interviews with key informants and pregnant or postpartum women were used to examine cessation needs, barriers to the provision and uptake of cessation support and directions for policy, practice and programming. Interviews were conducted with key informants from provincial organizations that offer cessation, maternal and/or child health support to women across Ontario. To maximize learning from different contexts and experiences, interviews were also conducted with key informants and women in three regions of Ontario. This included a major metropolitan area (Central) and two medium-sized, urban/rural mix areas (Central East and Northwestern). Key informants were individuals with insight into the needs of pregnant and postpartum women who smoke and/or who had expertise in the development, coordination or provision of cessation support to this population at the local and provincial levels. Inclusion criteria for women were: pregnancy status (pregnant or up to one year postpartum) and smoking status (current or former smoker or making a quit attempt). Written consent was obtained from all participants of the study prior to interviews. This study was approved by the Research Ethics Board of the University of Toronto.

### Recruitment and interview guide

#### Key informant interviews

Two researchers interviewed a total of 31 provincial and local level key informants (Table 1). The key informant interview guide explored perceived strengths of current cessation support for women, barriers and facilitators to service provision and ways to improve cessation support for this subpopulation. In consultation with the research team and the study’s advisory committee, the researchers purposefully invited a representative from relevant organizations to participate in an interview. In some instances, the researchers recruited key informants through snowball sampling [24]. Data collection took place between February and May 2011. Interviews were conducted over the phone and lasted between 45 minutes and one hour.

**Table 1:** Description of key informants (n = 31)

**Stakeholders**	**Represented organizations/professions**	**n**
Provincial key informants	Maternal addictions helpline	15
	Provincial smoking cessation helpline	
	Health professional governing bodies	
	Maternal/child health organizations for women and new families who experience disadvantage	
	Regional tobacco control coordinators	
	Resource centre for health providers who work with expectant families, newborns and young children.	
	Neonatal care	
	Academia	
Local key informants (Central, Central East and Northwest Regions)	Public health nurses from tobacco control and reproductive health departments	16
	Public health unit managers	
	Counsellors from organizations that serve pregnant and postpartum adolescents	
	Front line staff from Aboriginal, community health and addiction organizations that offer pre and postnatal programs	

#### Interviews with pregnant and postpartum women

The two researchers also conducted interviews with 29 women across the three public health regions between March and May 2011. The interview guide explored women’s smoking, quitting and relapse experiences, awareness and use of local and provincial smoking cessation support, barriers to access and suggestions to better help women address their tobacco-related goals. Participants were purposefully recruited by promoting the study through the provincial cessation helpline; a maternal, newborn and child health promotion network; and gatekeepers who work with the target population at local community agencies. Participants had the opportunity to participate in self-selected friendship pairs or individually [25]. Eighteen women opted to participate in the interview with a friend or acquaintance who also met the study’s inclusion criteria and the remaining 11 women participated as individuals. Interviews lasted between 30 minutes and one hour and the majority occurred in person at the local community agencies that served the women. Six interviews were conducted by phone. All participants received a $30 gift card to a pharmacy, which does not sell tobacco products, for participation. Demographic characteristics of women participants are found in Table 2.

**Table 2:** Demographic characteristics of women participants (n = 29)

	**n or mean**
**Age**	22.1 (range: 15–49)
Location*	
Central	13
Northwest	11
Central East	5
**Maternal status**	
Postpartum	19
Pregnant	10
**Ethnic or cultural roots**	
Aboriginal	11
White	11
Black	4
West Indian	1
Latin, Central or South American	1
Rather not say	1
**Education**	
< High school	21
High school	3
> High school	5
**Marital status**	
Has a partner	18
Single	11
**Living arrangement**	
Partner and child/children	9
Children only	7
Maternal residence	5
Parents	5
Partner and parents/other family	3

* While Central East and Northwest jurisdictions represent an urban/rural mix, all women, except for two (2), were living in an urban area at the time of interviews. Importantly, these participants’ accounts reflected a relatively transient life, and some women described living in rural/remote areas (i.e., fly-in, on-Reserve communities) at different stages of their lives.

### Analysis

All interviews were audio recorded and transcribed verbatim. To maintain data intimacy, interview transcripts were read by the researchers who conducted the interviews and discussed among the project team prior to coding. A thematic interpretive analysis was used, whereby transcripts were coded by the two researchers in NVIVO 9 using a coding structure based on the interview guide. Codes were subsequently organised by theme. Themes from interviews with key informants and women were then compared on similarity and contrast of content. This was repeated focusing upon contextual factors, such as geography, age and culture.

## Results

This section explores policy, practice and program-related barriers to the provision and uptake of services and initiatives to improve support for pregnant/postpartum woman (Table 3 and Table 4). The results are organized within three major themes: 1) Policy (approach and resources), 2) Program and Service Provision (approaches, consistency, engagement and accessibility), and 3) Contextual Factors (geography, stage of life, Aboriginal status). Extracts from interview transcripts are used to illustrate the themes and barriers, and are identified by stakeholder status (key informant, pregnant/postpartum woman) and region (Provincial, Central, Central East, Northwest).

**Table 3:** Perceived barriers and suggestions to improve cessation support for pregnant/postpartum women

**Theme**	**Barriers**	**Suggestions to address barriers**
**Policy**	Absence of provincial cessation strategy	Comprehensive cessation strategy that involves cross-sector and cross-ministerial collaboration
	Sustainable funding and organizational capacity issues	Funding for capacity building, program development and sustainability
		Organizational cessation polices, practices and procedures
	Hesitancy prescribing NRT to pregnant women	Medical directive for off-label use of NRT and education for providers
**Program and service provision**	Lack of comprehensive programming approach	Social determinants of health and harm reduction approaches
		Programs accessible through multiple sectors
		Address partner and family smoking
	Engagement and accessibility issues	Engage women in program development/implementation
		Incentives, transportation, childcare and snacks
		Program promotion through local venues
		Home visits
		Address stigma and misconceptions through:
		• Training health care providers to understand issues faced by women and role of smoking in lives
		• Cessation training for providers who work with disadvantaged women
		• Positive messaging
		• Provincial media campaigns
		Affordable NRT options
	Inconsistent provider practice	Annual training in MCI and cessation best practices for pregnant/postpartum women
		Perinatal cessation modules in educational curriculums/continuing education credits
		Knowledge exchange channels to facilitate patient referral and promote inter-professional learning

**Table 4:** Suggestions to address perceived barriers related to contextual factors

**Contextual factors**	**Suggestions to address barriers**
Rural/remote location	Resources and strategies to increase number and diversity of health/social care providers and to provide cessation specific training
	Transportation, peer telephone support or mobile health bus service
Adolescents	Greater provider sensitivity towards adolescents
	Incorporate teen programming into cessation/pregnancy resources
	Interweave tobacco and sexual health education/counseling
	Youth empowerment approach
Aboriginal women	Social welfare and access to education measures
	Adoption of Aboriginal health perspective

### Policy

#### Absence of a provincial smoking cessation strategy

The absence of a provincial smoking cessation strategy was identified as a major barrier, which has resulted in a perceived lack of coordinated cessation resources for not only pregnant and postpartum women, but for the general population as well.

*I think it’s very limited. I mean, they have access to everything the general public has access to but even the general public access is limited… sort of a patchwork of strategies that are loosely weaved together.* (Key informant, Provincial)

Key informants also perceived the cessation requirement outlined in the Ontario Public Health Standards as vague. As some noted, simply promoting the provincial cessation helpline to clients meets this requirement. This reportedly contributed to the low dedication of public health funds to cessation compared to prevention and protection initiatives, ultimately limiting the provision of local cessation services.

A comprehensive cessation strategy was thus recommended to facilitate coordination of cessation resources at the provincial and local levels and improve the availability, accessibility and effectiveness of support. The need for cross-sector and cross-ministerial collaboration for smoking cessation policy and programming, especially in relation to special populations, was also highlighted. Key informants especially emphasized the importance of integrating tobacco-focused policy, practice and programming into reproductive and child health fields. As one key informant articulated, policy that addresses smoking during pregnancy should be, “*a pillar of any maternal and child health strategy.*” Further, affordable Nicotine Replacement Therapy (NRT) was considered a necessity to establishing a comprehensive system. In addition to advocating for more education for practitioners and the public about the perinatal use of NRT, the need for policy-level change was clear.

*…if there were a Chief Medical Officer of Health statement, or dictum or policy that comes down that says, ‘We fully support the use by prenatal women of nicotine replacement under recommendation from pharmacists,’ that would go a long way to providing additional support and services.* (Key informant, Provincial)

Consistent with a comprehensive approach, key informants broadened their recommendations to include polices beyond cessation. Participants identified easy access to cigarettes and exposure to other smokers and secondhand smoke in apartments as barriers to cessation. Informants thus highlighted the need for greater taxation of tobacco products, measures to reduce the availability of cheap cigarettes and further development of smoke-free policies (i.e., smoke-free multi-unit dwellings). Others centered primarily on targeting the social determinants of health, such as poverty reduction, housing and education support.

*…more education, more access to social programs…let’s address the gap of teen services, address poverty issues in general in that the federal policies could create more employment because…it is a statistical fact that there is more smoking in the lower income level of the population. So lower the cost of education and the barriers to education.* (Key informant, Provincial)

#### Sustainable funding and organizational capacity issues

A lack of sustainable funding was highlighted as a major barrier to the reach and provision of cessation services for this subpopulation. One key informant, a health counselor who works with pregnant and postpartum adolescents, expressed frustration with her inability to access resources and tools due to budgetary issues.

*We don’t have the resources, we don’t have the clinicians, we don’t have the tobacco replacement system…We don’t have any of those. So we have to send them out, if there is anything available outside.* (Key informant, Central)

This key informant identified a lack of programs and practitioners specialized in smoking cessation as an additional barrier and expressed a desire for a dedicated program within their organization. Funding issues also restricted capacity to reduce barriers to access (i.e., transportation and childcare) and exchange knowledge with other organizations and service providers. In the same vein, a lack of staff time and resources to develop and support services and programs was also highlighted, especially within hospitals, public health units and local community agencies. Importantly, capacity issues were not limited to the tobacco control community but to the reproductive, child and mental health communities as well. Key informants highlighted the need for organizations to adopt cessation policies, practices and procedures that focus on working with pregnant and postpartum women who smoke and felt that more funding would allow service providers to continue to build capacity within the community and eventually reach more pregnant and postpartum women. The need for sustained funding was thus mentioned several times in different contexts: to build a coordinated cessation system, to build the workforce in perinatal smoking cessation and to ensure the development and continuity of programs during and after pregnancy.

### Program and service provision

#### Lack of a comprehensive programming approach

There was a perceived absence of cessation programs tailored to meet the diverse needs of women. Specifically, programming lacked a woman-centred approach that incorporated the social determinants of health. Key informants also noted that existing cessation programs were *“too cessation-focused”* and emphasized the importance of adopting harm-reduction approaches, specifically reducing consumption, when working with this population. Harm-reduction was a means to build self-efficacy in the pursuit of quitting and reduce the fear and/or feelings of failure should a woman not meet a quit date or relapse. Key informants thus advocated for a “*no-wrong door*” approach to cessation. This entailed having dedicated cessation programs for pregnant and postpartum women within public health and integrated support in maternal and child health services. In line with this suggestion, both women and key informants stressed that smoking by partners, family members and friends deterred quit or reduction attempts and thus conveyed the need for programs and services that target partner and family smoking.

*It’s hard because I want to keep trying to not do it all but it’s just always in my mind. Yeah, and I see him doing it all the time…I’m glad now that we’re living at in his dad’s that it’s an outside thing. You have to go outside to smoke because the place we’re living in before, everyone is smoking inside. It was horrible.* (Pregnant woman, Central region)

#### Inconsistent practice

Key informants sensed a lack of comfort and confidence in addressing smoking cessation among service providers and a lack of clarity about the safety of perinatal cessation and the use of NRT/quit smoking medication. Some acknowledged that providers sometimes feel uneasy addressing tobacco and other addictions in this population and that fear of harming the fetus through smoking cessation activities might be the root cause of this trepidation.

*…I find, fear and stigma around working with pregnant women because of (Name of Children’s Interest Organization), because there is this unborn child and, and there’s that sort of stuff that you can’t…like it’s hard to control for everything.* (Key informant, Central region)

Further, women in this study reported discrepancies in the cessation support received from their providers. Some providers reportedly advised women to cut down and eventually quit and continued to inquire about smoking throughout the pregnancy. Others reportedly inquired at the beginning, but did not follow-up throughout pregnancy. Women also described direct and indirect experiences with providers’ hesitancy prescribing NRT.

*They told me here not to do that and they wouldn’t give me the patch because I was pregnant.* (Postpartum woman, Central region)

Key informants recommended annual training in minimal contact intervention (MCI) and training in the best practices in smoking cessation for pregnant and postpartum women, as well as incorporating modules on perinatal cessation into diploma, undergraduate, postgraduate or continuing education credits. Developing systemic relationships between organizations, practitioners and experts that work with this population was also suggested to facilitate referral within and between community organizations, promote individualized treatment plans and promote learning across sectors and communities. Knowledge exchange channels, such as perinatal smoking cessation coalitions, communities of practice and/or working groups were recommended to achieve this.

#### Engagement and accessibility issues

The women noted competing priorities as a barrier to engaging in cessation support. Attending pre/postnatal appointments, alternate substance use, difficult domestic situations and socioeconomic factors, such as housing and food insecurity, often take precedence in these women’s lives, potentially leaving little time to attend a cessation program or service. Women also highlighted the following as barriers to accessing programs: failure to meet program inclusion criteria, inadequate cessation support for partners and family members, lack of childcare and transportation, timing of programs, cost and fear of failure.

Participants highlighted various ways to improve reach and engagement. Engaging local women in program development and implementation was important to inform relevant and accessible community support, establish program ownership and thus facilitate uptake. Providing incentives such as grocery or pharmacy gift certificates or free meals was considered especially useful for pregnant and postpartum participants who are more socioeconomically disadvantaged. For example, a young mother discussed how the weekly grocery store gift cards she received for attending the local perinatal cessation group empowered her to replace smoking with healthy eating.

*They give free transportation and they give you a $20.00 gift card towards (Name of grocery store) to get your fruits or whatever you need to supplement healthy eating and to stop binge eating from smoking [Laughs]…I think it’s a really good incentive for people to go…I feel good about myself and this is making me feel even better because after this I don’t need a cigarette. I can go to the grocery store and get healthy food or whatever.* (Postpartum woman, Central East Region)

Key informants recommended offering transportation, childcare and meals/snacks; adopting woman-centred and harm-reduction approaches; and promoting programs through a variety of local venues (i.e., grocery store bulletin boards, laundromats, community centres). Provider home visits were also perceived as an avenue to better understand the clients’ needs and their environment, to tailor support and build provider-family trust.

In addition to program characteristics, stigma and misconceptions about smoking cessation during pregnancy emerged strongly in interviews and were noted barriers to accessing adequate support. Women often mentioned hiding their smoking and their bodies because of the judgment they experienced when they smoked in public or in front of family. While some women felt very comfortable with their physicians, others felt intimidated or feared judgment. A young mother described feeling *‘ashamed’* and *‘devastated’* when ‘*people stared’* and when her physician spoke to her about smoking during pregnancy. Key informants believed that stigma and fear of judgment prevented some women from revealing smoking status to their providers and/or from accessing help. This was evident in the narratives of some women.

*I find like I’m embarrassed and like if I go into the hospital or anything, I kind of like lie and say I don’t smoke, when I do, because I get less shamed when…they start like lecturing me. I get kind of frustrated and I just want it to be over and done with…So now if I go to like the hospital or if I go my appointments and stuff, I kind of lie and say I don’t smoke when I do…Which I shouldn’t do, but I don’t want to feel that judgment.* (Pregnant woman, Central Region)

Many women perceived quitting during pregnancy as too stressful for the fetus, potentially causing harm or even miscarriage. Some cited service providers and others cited family and friends as the source of this information. There was also confusion about the general safety of using the nicotine patch and its effects not only on the woman’s health, but the health of the baby. When asked about the nicotine patch, a young pregnant woman noted that she “*didn’t think pregnant woman could take them.*” Two young women felt that NRT was “*not good for you*” because it “*screws up your period,*” and “*screws up your sleeping patterns.*” These young women likened NRT to *“24/7 cigarette.*” Key informants vocalized concern that misconceptions might prevent women from seeking help and/or providers from offering appropriate support.

To address issues surrounding stigma and misconceptions, key informants articulated the importance of training service providers to understand the role of smoking within a woman’s life, use positive messaging and to practice in an empathetic manner. In addition to physicians and public health nurses, key informants emphasized the need for smoking cessation training among health/social care professionals who work with women, particularly those who work with women that experience disadvantage. Social, youth, addiction and community health workers were considered especially important as they often have longstanding trusting relationships with women. Finally, key informants conveyed the importance of provincial media campaigns to raise awareness about programs and services, reduce stigma, and dispel the misconceptions surrounding quitting and NRT use during pregnancy.

### Contextual factors

#### Geographical issues

Key informants from Northern and rural communities perceived an inadequate allocation of funding for tobacco control and cessation activities relative to urban, larger communities. This reportedly extended to other areas of public health as well.

*…we have a background of less resources; less resources in every level, specifically within north-western Ontario.* (Key informant, Northwest Region)

This was of concern given the higher rates of maternal smoking in Northern and rural communities and the greater proportion of Aboriginal groups who live in these regions. Further, geographical dispersion of residents posed additional barriers to reach and engagement. A postpartum woman described how this impeded her participation in a new cessation support group for pregnant and postpartum women:

*It would be easier if we could go as a family…with (Name of partner), because he’s my wheels [Laughs]. We live in (Name of town) and he’s got to drive 25 minutes into town to take me somewhere…He’s not going to drive all the way back home and then come and get me.* (Postpartum woman, Central East Region)

Women and key informants thus echoed the importance of offering transportation to overcome barriers related to geographic dispersion. Similarly one key informant recommended a peer-to-peer service whereby women with smoking experience call other women to offer cessation support, while another described the possibility of creating a service that utilizes a “mobile health bus” to reach individuals who live in remote communities.

Recruitment and retention of health human resources were also perceived barriers that limited the ability to provide sustainable support in more remote locations. Interestingly, women from the metropolitan location described cessation experiences with providers beyond their primary physician. These included dieticians, midwives, sexual/reproductive health nurses, counsellors and/or social workers. Generally, other health professionals consistently encouraged these women to cut down and eventually quit, for which the women were appreciative. Contrastingly, women from the smaller, urban/rural mix communities only spoke about their primary physician or nurse practitioner. Key informants noted that additional resources and strategies are needed to increase the number and diversity of health/social care providers, provide cessation-specific training and consequently increase the availability of cessation support in rural/remote communities.

#### Adolescent mothers

Stigma appeared to emerge more strongly among the younger women in this study, reflecting the need for greater sensitivity among service providers who work with adolescents. Similarly, adolescents frequently failed to disclose smoking status to their physicians at the beginning of their pregnancy because they considered themselves already quit. Thus, physicians might have viewed their patients as non-smokers, possibly compromising the provision of support. Adolescents were also less enthusiastic about group programs due to a fear of not getting along with other participants and/or the fear of being judged by participants and service providers.

Several informants highlighted the absence of targeted programming for teen mothers in traditional smoking cessation programming and in general pregnancy resources. Adolescent mothers were considered a vulnerable population where supports and incentives might play an even bigger role since they are more likely to experience financial insecurity and to be without a partner. The importance of offering such services was also evidenced by the gratitude the younger women expressed for the transportation and childcare they received to attend their current maternal health programs. Given the population, schools were suggested as a natural environment to reach adolescents. A few key informants believed that integrating tobacco into sexual health education and counselling might help address teen pregnancy and teen smoking simultaneously. Others stressed a stronger approach to targeting youth in order to prevent smoking uptake. This entailed a youth empowerment approach that focuses upon improving resiliency amongst youth and young adults, especially during key transitional stages.

*…we also need to talk about resiliency and arming yourself against bad behaviours or false behaviours as we go from high school into the first job, high school into college or high school into university. There is another time when the smoking uptake really continues to climb and stays at that level in the early 20’s and that influences the climate during those early childbearing years.* (Key informant, Northwest Region)

#### Aboriginal women

Key informants highlighted inadequate funding to meet the unique cessation needs of Aboriginal women. Funding issues were also raised in the context of social welfare and access to education measures, which some perceived as key to breaking the cycle of disadvantage that encourages smoking uptake and persistence in Aboriginal communities. This was especially the case for Aboriginal women who leave fly-in communities to pursue high school education in larger cities.

*…so we have several hundred students who, to continue their high school education, are housed with host families in (Name of city). Around grade nine to grade twelve a tremendous number of them still drop out. So the young women go home and then they have a couple of kids. Meanwhile they live on social assistance; then they try and pick up some future employment through college education …meanwhile the federal level has been cutting back on educational entitlements; we have the fact that there’s less per capita spent by the federal government on the schools in the north. So you have individuals who have a number of disadvantages and then you throw in also being pregnant and of course you continue your disadvantaged life.* (Key informant, Northwest Region)

Key informants articulated the importance of adopting an Aboriginal health perspective into programs and services. This perspective was described as one that addresses traditional tobacco use and adopts a holistic approach, which involves the physical, emotional, mental and spiritual characteristics of a person as well as one’s family and community. Aboriginal women in this study did not identify barriers to accessing services. This was likely the case because they were already provided with support to access their maternal and child health programs and services. However, scheduling conflicts associated with children being ill and out of school/daycare was common.

## Discussion and conclusion

### Cessation support for pregnant and postpartum women

The cessation system in Ontario is not equipped to adequately reduce smoking among pregnant and postpartum women. Inadequacy in support reflects the absence of a provincial cessation strategy, which has impacted not only support for pregnant and postpartum women, but for the general smoking population as well [18]. A comprehensive system would set the stage for a multi-sector initiative designed to provide tailored, sustainable support to women that is accessible through multiple system entry points.

This study also contributes to the general knowledge about how pregnant and postpartum women who smoke relate to cessation services and how providers relate to service provision for this population. Specifically, it points to variability in provider practice around cessation for pregnant and postpartum women, which is consistent with previous findings [22,23]. A cultural shift in practice is thus necessary to eliminate mixed messaging, and encourage open channels of communication about smoking between women and their providers. Due to the stigma associated with smoking during pregnancy, engaging providers who already have relationships with women and who are in a position to offer continued support is equally necessary. This is important because smoking is often perceived as a coping mechanism for stress and the stress associated with feelings of guilt and shame could potentially reinforce smoking. Further, failure to disclose smoking status or seek help also poses implications for monitoring and surveillance of smoking rates. Capacity building strategies that focus on strengthening providers’ competency and confidence in practice and that encompass training and education for providers who play significant roles in reproductive and child health are needed. While this study did not focus upon specificities of training and educational strategies, some initiatives that have targeted organizational capacity and health care provider practice have shown success in improving short-term knowledge and engagement in perinatal cessation [22]. Further research is necessary to inform training and educational strategies.

### Considering the context

In Ontario, geographical features, such as remoteness/rural location and shortages in health human resources present significant challenges to engaging a population already considered very difficult to reach. Future research should examine strategies that aim to improve the provision of perinatal cessation support in rural and remote locations (i.e., extending provider incentive programs to health and social care professionals beyond physicians [26,27]) and how best to address barriers related to geographic dispersion/isolation. Further, the elevated rates of smoking in pregnancy among adolescents [17] and Aboriginal women in Canada [28] exemplify the need for culturally sensitive practices and tailored interventions. Elevated pregnancy smoking rates are also found among women who experience mental health and addiction issues and/or traumatic life events [29]. As emphasized by Greaves and colleagues, future research should explore the smoking and cessation experiences in pregnancy by subpopulation and evaluate the effectiveness of tailored strategies [29].

### Cessation and beyond: adopting a comprehensive approach

This study also highlights the need to address smoking among women in a more holistic manner. Tobacco control policies in the realm of prevention and protection contribute to creating environments that facilitate the cessation process [30-32]. Specifically, easy access to cigarettes and exposure to smoking in common areas and private units in apartments were noted barriers to smoking cessation. Thus, policy that addresses the availability of cigarettes, and restricts exposure to SHS in multi-unit housing emerged from our data as areas requiring further development. Importantly, research suggests the emergence of unintended consequences of tobacco control policies on women who experience disadvantage. This includes increased stigmatization from interventions designed to reduce children’s exposure to secondhand smoke [33] and increased financial stress due to tobacco taxation [34]. Evaluation that employs gender based analysis techniques [33] is thus essential to understanding and addressing the unintended impacts of policies on smoking behaviour of expectant and new mothers.

It is also suggested that women are often extrinsically motivated to quit and a shift in identity from smoker to non-smoker does not occur [29], potentially contributing to high postpartum relapse rates. A women-centred approach contextualizes the cessation experience within the unique social worlds of women and emphasizes quitting for the woman’s health rather than only that of the fetus/child. Due to its focus on increasing intrinsic motivation, such an approach might contribute to reducing relapse. Further, partner smoking is an important predictor of a woman’s smoking status [35] and many adolescents and young adults live with parents and/or extended family members who smoke. Research and interventions that address smoking among partners and other family members are necessary.

Finally, this study highlights a major health inequity within Ontario and the importance of addressing the social determinants of health that shape smoking behaviour before, during and after pregnancy. The literature demonstrates that pathways of disadvantage throughout the lifecourse shape the smoking behaviour of women [36,37]. Specifically, research from the United Kingdom demonstrates that poor childhood and adult socioeconomic circumstances, educational disadvantage, being a young mother and a single parent each increase a woman’s risk of being a smoker before pregnancy. Childhood socioeconomic circumstance, education and adult socioeconomic circumstance are also powerful predictors of quitting in pregnancy [38]. Thus, an upstream approach to tackling the ‘causes of the causes of ill health’ is needed [39]. Graham and colleagues suggest that the tobacco control community complement its focus on changing smoking behaviour with policy that seeks to moderate the social conditions that shape smoking (i.e., social welfare measures, educational policy) [13,37]. Given that tobacco use often clusters within socioeconomically disadvantaged populations, this approach might be fruitful among other populations as well.

### Study strengths and limitations

The findings from this study are limited to the three public health regions in Ontario where data was collected. However, data were triangulated between provincial and local informant interviews, interviews with women and with the literature to reveal consistent and unique findings. In addition, the stigma associated with smoking during pregnancy may have elicited more normative responses, which could have reduced the depth of qualitative data achieved. To minimize this, participants were given the opportunity to participate in an interview with a peer, the interview guide was developed to create an atmosphere conducive to talking openly and, when possible, interviews took place either at a community agency familiar to the women or over the phone. Further, the data was collected by two young adult females with previous experience conducting interviews with sensitive populations. Despite these limitations, this study captured the complexity of key informants’ experiences in the development, implementation and provision of cessation support, and of pregnant and postpartum women’s experiences with quitting and use of cessation support. This study is unique in that it contributes to the adoption of a comprehensive approach to improving support for women, examines contextual factors that shape the uptake and provision of service and programs and explores the perspectives of a diverse sample of stakeholders.

## Endnotes

^a^The Ontario Public Health Standards establish the minimum requirements for public health programs and services to be delivered by Ontario’s 36 public health boards. Requirement 9, the cessation requirement, stipulates: “*The board of health shall ensure the provision of tobacco use cessation programs and services for priority populations*.”

## Competing interests

The authors declare that they have no competing interests.

## Authors’ contributions

Study design: AB, RS, TB: data collection: TB, SI; analysis and interpretation of the data: TB, SI, AB, Manuscript preparation: TB, AB, RS SI. All authors read and approved final manuscript.

## Pre-publication history

The pre-publication history for this paper can be accessed here:

http://www.biomedcentral.com/1471-2458/13/472/prepub
